# Editorial: Recent advances in the molecular genetics and precision medicine of lung carcinoma

**DOI:** 10.3389/fgene.2024.1369247

**Published:** 2024-01-22

**Authors:** Mehdi Pirooznia, Xiaogang Wu, Jonathan N. Bella, Fan Zhang, Dragana Jovanovic

**Affiliations:** ^1^ Johnson & Johnson, Washington, DC, United States; ^2^ The University of Texas MD Anderson Cancer Center, Houston, TX, United States; ^3^ Icahn School of Medicine at Mount Sinai, New York, NY, United States; ^4^ University of North Texas Health Science Center, Fort Worth, TX, United States; ^5^ Policlinic Akta Clinica, Belgrade, Serbia

**Keywords:** lung cancer, adenocarcinaoma, NSCLC, LUSC (lung squamous cell carcinoma), LUAD (lung adenocarcinoma)

The goal of this Research Topic is to elucidate the latest research in lung cancer genomics and clinical advances. Lung Carcinoma is the second most common cancer worldwide, with more than 2.2 million cases recorded globally in the year 2020, as well as 1.8 million deaths. It is the most common cancer in men and the second most common cancer in women, with an estimated lifetime risk of developing the disease of 1 in 15 for men and 1 in 17 for women. Whilst being the most prevalent cancer, it is important to note that smoking is the single biggest risk factor for developing lung cancer, with over 70% of cases being estimated to have been caused by tobacco smoke, and excessive smoking potentially leading to as much as a 25-fold increase in the likelihood of the disease developing. Recent advances in sequencing technology, computational approaches, and our biological understanding of lung cancer have revolutionized how we diagnose, prognosticate, and treat lung carcinoma. Genetic studies into this disease have revealed a plethora of information which can be used to combat the cancer, such as novel biomarkers and gene signatures, as well as opening the door to more of a “personalized medicine” approach.

We hope that this Research Topic will inform, inspire, and provide guidance to researchers in the field. Here is an overview of the issues presented in this Research Topic.

The clinical significance of ferroptosis in lung adenocarcinoma (LUAD) was investigated by Ren et al. They constructed a 15-gene prognostic signature predicting overall survival based on ferroptosis-related in The Cancer Genome Atlas (TCGA)-LUAD cohort. Functional analysis revealed that ferroptosis was closely related to cell cycle, cell metabolism, and immune pathways. This signature also acts as a regulator modulating drug resistance, tumor microenvironment infiltration, and cancer stemness. Furthermore, genes in this signature were screened as potential novel immunotherapy biomarkers targets for further research as their biological functions in ferroptosis were consistent with their prognostic significance. In another related study, Chai et al. studied ferroptosis antitumor immune responses in LUAD. They identified 38 ferroptosis-related (FRGs) and 429 immune-related (IRGs) differentially expressed genes between tumor and normal samples and developed Lasso-penalized Cox regression risk score formulas for them. The correlation between FRGs and IRGs was evaluated using the TIMER database. The outcome indicated that the development of ferroptosis was synergistic with that of anti-tumor immunity which may be useful for future investigation of prognostic value and therapeutic potential related to ferroptosis and tumor immunity in LUAD. Zhang et al. also studied Fanconi anemia group D2 (FANCD2) which is a ferroptosis-related gene crucial for DNA damage repair and negative ferroptosis regulation. They evaluated FANCD2’s association with ferroptosis and immune infiltration in LUAD using transcriptome sequencing data, clinical information, and immunohistochemistry data were collected from the TCGA, GEO, and HPA databases, respectively, for three independent cohorts. Univariate and multivariate analyses were used to assess the correlations between FANCD2 expression and overall survival or clinicopathological parameters. Their analysis revealed that FANCD2 expression levels were significantly related to tumor-infiltrating immune cells and their matching gene signatures, including CD8^+^ T cells, natural killer (NK) cells, dendritic cells (DC), and Th2 cells in cases of LUAD and that FANCD2 is a crucial molecule underlying the synergistic effects of ferroptosis and immunotherapy for Patients with LUAD.

Chen et al. investigated the diagnosis and prognosis roles of DNASE1L3 gene in LUAD. They reported that low DNASE1L3 expression was significantly associated with higher pathological stages, T stages, and poor prognosis in LUAD cohorts and indicated that the mRNA level of DNASE1L3 was positively correlated with the infiltration of various immune cells, immune checkpoints in LUAD, especially with some m6A methylation regulators. Moreover, they reported that DNASE1L3 was positively related to G protein-coupled receptor ligand biding and G alpha (i) signaling events. Another study conducted by Zhao et al. aimed to identify the key intercellular communication-associated genes (ICAGs) in LUAD. They identified prognosis-related ICAGs and developed a risk score by using survival analysis in TCGA-LUAD. Machine learning models were then trained to predict LUAD recurrence based on the selected ICAGs and clinical information as well as comprehensive analyses on ICAGs and tumor microenvironment. The model achieved a remarkable area under receiver operator characteristic curves of 0.841. DNA replication and cell cycle were significantly enriched by the differentially expressed genes between the high- and the low-risk groups according to their risk scores. The study identified eight key ICAGs in LUAD, which could contribute to patient stratification and act as novel therapeutic targets.

The PD-1/PD-L1 inhibitors, widely used in clinical trial, showed a low response rate and were effective for only a small number of cancer patients. Mao et al. investigated the low response rate of PD-1/PD-L1 inhibitors immunotherapy. They performed ssGSEA and unsupervised clustering analysis to identify clusters according to different immune cell infiltration status, prognosis, and biological action. One cluster showed a better survival rate, higher immune cell infiltration, and immunotherapy effect, with enrichment of a variety of immune active pathways including T and B cell signal receptors. Their analysis suggested that there were six genes with KLRC3 as the core which can efficiently improve immunotherapy responses with greater efficacy and better prognosis.

There is accumulating evidence that long noncoding RNAs (lncRNAs) are playing critical role in predicting the prognosis and immune response in carcinoma including LUAD. Using cooexpression analysis in LUAD patients, Peng et al. identified mitochondrial homeostasis–related lncRNAs (MHRlncRNAs) and mitochondrial homeostasis–related lncRNA signature (MHLncSig) as an independent predictive factor of prognosis. The study further investigated the underlying tumor microenvironment, tumor mutation burden, and immune landscape behind different risk groups and suggested that MHLncSig may be a promising tool for predicting the prognosis and guiding individualized treatment in LUAD. The lncRNAs are also investigated in lung squamous cell carcinoma (LUSC) by Huan et al. They screened the TCGA-LUSC samples and constructed a 5-lncRNA-based signature, combining lncRNA and traditional clinical indicators for prognosis prediction. The signature was significantly related to chemotherapy response, especially in cisplatin, vinorelbine, and paclitaxel. The enrichment analysis indicated that co-expression mRNAs of the 5 lncRNAs were mainly focused on RNA splicing, DNA replication, and protein serine/threonine kinase activity. Functional assays also demonstrated regulated invasion, migration, proliferation, and programmed death *in vitro*. They concluded that the 5-lncRNA-based signature has a good performance in predicting immune characteristics and prognosis of LUSC patients. On another related study, Zhang et al. investigated the interactions between ferroptosis and lncRNAs for LUSC, and its impact on tumor immune microenvironment. They identified a ferroptosis-related lncRNAs signature (FerRLSig) for LUSC prognosis and evaluated its correlation to tumor immune evasion. Based on the FerRLSig stratification, the high-risk group demonstrated significantly higher immune infiltration, as well as more severe T cell dysfunction and immune evasion, which might ultimately lead to the resistance to current immune checkpoint inhibitors. Tetik Vardarlı et al. evaluated whether lncRNAs obtained from exhaled breath condensate (EBC) samples play a role in the occurrence of metastasis in the diagnosis and follow-up of patients with advanced LUAD. They observed lncRNAs HOTAIR, PVT1, NEAT1, and MALAT1 expression levels were significantly higher than those in healthy controls. They proposed that EBC can be used as an innovative and easily reproducible approach for predicting the development of metastases, molecular diagnosis, and follow-up of lung cancer.

Tang and Guo studied dysregulation of the ubiquitin-proteasome system (UPS) that can lead to instability in the cell cycle and may act as a crucial factor in both tumorigenesis and tumor progression. They retrospectively evaluated a total of 703 LUAD patients through multivariate Cox and Lasso regression analyses, and developed an eight-UPSG signature, including ARIH2, FBXO9, KRT8, MYLIP, PSMD2, RNF180, TRIM28, and UBE2V2 as a novel prognostic predictor for LUAD. On another study, Wang et al. employed ssGSA, WGCNA, univariable and LASSO Cox regression analyses of LUAD patients to build a fatty acid-related risk score (FARS) model. They established a nomogram based on the FARS and other clinicopathological features and used ROC and calibration plots were used to validate the prediction accuracy. The tumor microenvironment (TME) of patients with high and low FARS was compared. And a total of 38 genes were identified to be independently related to the survival outcome and put into a FARS model. High FARS patients exhibited significantly worse OS. Patients with high FARS can potentially benefit more from anti-PD1/PDL1 immunotherapy.

Zhao and Lu investigated clinical significance and prognostic and immunological function of 26S proteasome non-ATPase regulatory subunit 2 (PSMD2) in TCGA-LUAD. They conducted PSMD2-related immune infiltration analysis and tumor-Immune system interaction database (TISIDB) to verify the correlation between PSMD2 expression and tumor-infiltrating lymphocytes (TILs). They observed that both mRNA and protein expression of PSMD2 were significantly elevated in LUAD. High expression of PSMD2 was significantly correlated with high T stage, lymph node metastases, and TNM stage. The genetic mutation of PSMD2 was also correlated with poor overall survival, disease-specific survival, and progression-free survival in LUAD. Functional enrichment suggested PSMD2 expression was involved in the cell cycle, RNA transport, and cellular senescence suggesting that PSMD2 is a potential biomarker for poor prognosis and immune therapeutic target in LUAD.

Abnormal chromosome segregation regulators (CSRs) is a common hallmark of cancer. Li et al. studied the specific predictive value of it in LUAD. They performed unsupervised clustering to identify the distinguishing clusters in LUAD patients based on the expression of CSRs. The immune environment estimation, including immune cell infiltration, HLA family genes, immune checkpoint genes, and tumor immune dysfunction and exclusion (TIDE), was then assessed between the clusters. Cell cycle and chromosome segregation regulated genes were enriched in cluster 1, while metabolism regulated genes were enriched in cluster 2. Patients in cluster 2 illustrated a higher score of immune, stroma, and HLA family components, while those in cluster 1 had higher scores of TIDES and immune checkpoint genes. They concluded that the CSRs were correlated with the poor prognosis and the possible immunotherapy resistance in LUAD.

Pyroptosis is a highly inflammatory mode of regulated cell death and essential for the remodeling of tumor immune microenvironment and suppression of tumor development. Zhang and Liu studied pyroptosis-related gene polymorphisms in non-small cell lung cancer (NSCLC). They genotyped six SNPs in the GSDMB, GSDMC, and AIM2 in 650 NSCLC cases and 650 healthy controls using a MassARRAY platform. Their findings showed associations with risk of NSCLC and provided new insights into the roles of pyroptosis-related genes as new factors for assessing the risk of development of NSCLC. In another study, Zhou et al. aimed to elucidate the relationship between genomic alteration and pseudoprogression (PsPD)/hyperprogressive disease (HPD) in immunotherapy-treated advanced NSCLC and to provide clinical evidence for distinguishing between PsPD and HPD. They selected patients with advanced NSCLC who were treated with anti-PD1 and performed Whole blood next-generation sequencing analysis (NGS). They reported the gene mutation profiling of PsPD and HPD differed before treatment. They indicated true disease progression in patients with HPD and suggesting dynamic whole-genome mutation profiling can distinguish PsPD from HPD more effectively and it can potentially be used as a novel method for guiding clinical immune treatment.

